# Separation and identification of indene–C_70_ bisadduct isomers

**DOI:** 10.3762/bjoc.12.88

**Published:** 2016-05-06

**Authors:** Bolong Zhang, Jegadesan Subbiah, David J Jones, Wallace Wing Ho Wong

**Affiliations:** 1 School of Chemistry, Bio21 Institute, University of Melbourne, 30 Flemington Rd, Parkville, Victoria, 3010, Australia

**Keywords:** chromatographic separation, electron acceptor, fullerene bisadduct, organic solar cell, regioisomers

## Abstract

Following an initial work on the isolation of a single geometric isomer from an indene–C_70_ bisadduct (IC_70_BA) mixture, we report the full fractionation and identification of the bisadduct species in the material. Eleven fractions of IC_70_BA isomers were separated by high-performance liquid chromatography. A number of fractions contained relatively pure isomer species and their configuration were deduced using a variety of analytical techniques including ^1^H and ^13^C NMR and UV–vis spectroscopy. The electrochemical properties and the organic solar cell device performance were investigated for fractions where a reasonable quantity of sample could be isolated.

## Introduction

Organic solar cells (OSCs) are an emerging renewable energy technology that has achieved remarkable progress over the past two decades. Compared to traditional inorganic semiconductor solar cells, OSCs promise a number of advantages, such as lightweight flexible devices and low-cost fabrication using roll-to-roll printing [1]. Bulk-heterojunction organic solar cells (BHJ OSC) are a specific type of OSCs which contain a blend of organic electron donor and acceptor materials as the photoactive component.

**Figure 1:**
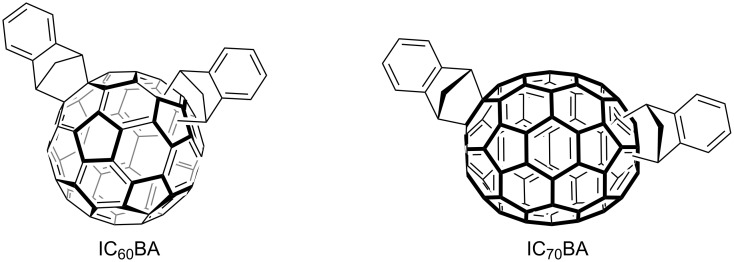
Molecular structure of IC_60_BA and IC_70_BA.

Fullerenes and their derivatives are widely used in BHJ OSC devices as the electron acceptor material. They have several characteristics that make them favorable for this application including good electron transport [2], reversible reduction behavior [3], and easily functionalized structures [4]. Indene fullerene bisadducts, specifically the C_60_ (IC_60_BA) and C_70_ (IC_70_BA) analogues (Figure 1), have been used successfully to boost the performance of poly(3-hexylthiopehene) (P3HT) based devices. The use of fullerene bisadducts improves the open circuit voltage of the device compared to mono-functionalized derivatives. In recent studies, the solar cell devices achieved power conversion efficiency as high as 7.5% for IC_60_BA [5] and 7.4% for IC_70_BA [6].

**Figure 2:**
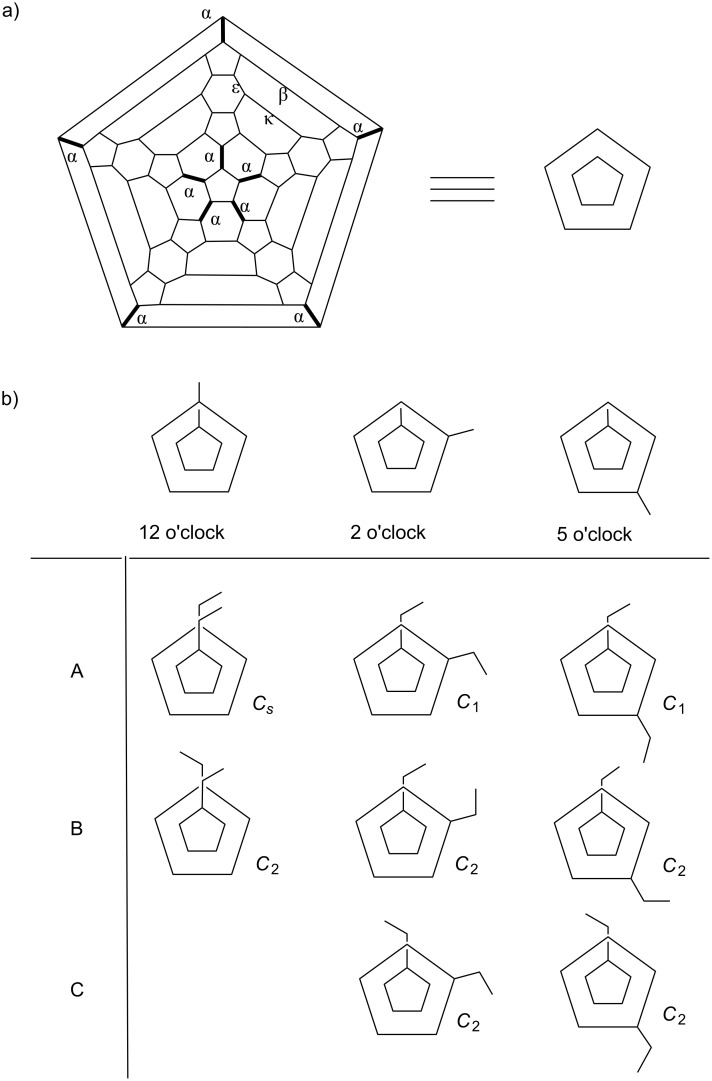
a) Schlegel diagram of C_70_; b) illustrations of three regioisomers of IC_70_BA and their geometrical isomers.

The IC_70_BA material used in most reports consisted of a mixture of isomers [7–9]. The synthesis of IC_70_BA involves [2 + 4] Diels–Alder cycloaddition reaction between C_70_ and two isoindene molecules generated in situ from indene. The symmetry of the ellipsoidal C_70_ molecule means that there are four different bonds between two six membered rings ([6,6]-bonds) that can participate in the Diel–Alder reaction. These are known as α-, β-, ε- and κ-bonds (Figure 2a). The α-bonds are the most reactive as they situated at the ends of the C_70_ molecule and therefore experience higher strain from the curvature of the molecule [10]. While reaction at non-[6,6]-bonds are possible, the thermodynamic products of fullerene adducts are usually located on the [6,6]-bonds [11]. Therefore, bisadducts of C_70_ usually consist of three major regioisomers, which have been described as the 12 o’clock, 2 o’clock and 5 o’clock isomers (Figure 2b) [10]. Each of these three regioisomers also includes two or three geometric isomers as a result of the conformation of the substituents.

Given the numerous possible isomers in IC_70_BA, our group took interest in examining the possibility of isolating single isomers from the mixture. It was anticipated that material containing a single isomer would have higher crystallinity compared to the mixture and this would have significant effects on device performance. In a previous communication [9], an isomer of IC_70_BA was obtained by chromatographic separation using both flash chromatography and high pressure liquid chromatography (HPLC). X-ray crystallography revealed that this sample contained the 2 o’clock-B isomer (Figure 2b). This material was used with P3HT in solar cell devices that showed higher performance compared to devices containing the isomer mixture. Analysis of the materials and devices indicated that the single isomer had better charge transport properties probably as a result of higher crystallinity of the material.

It is noteworthy that there are a handful of other studies in the literature that reported on chromatographic separation of fullerene bisadduct isomers [8,12–14]. To the best of our knowledge, this is the first comprehensive analysis of IC_70_BA mixture using HPLC. Eleven fractions were collected and analyzed. Among these fractions, all of the major regioisomers as well as some minor regioisomers of IC_70_BA were identified. The separation process, full characterizations as well as the device performance of these isomers of IC_70_BA are presented in this work.

## Results and Discussion

**Figure 3:**
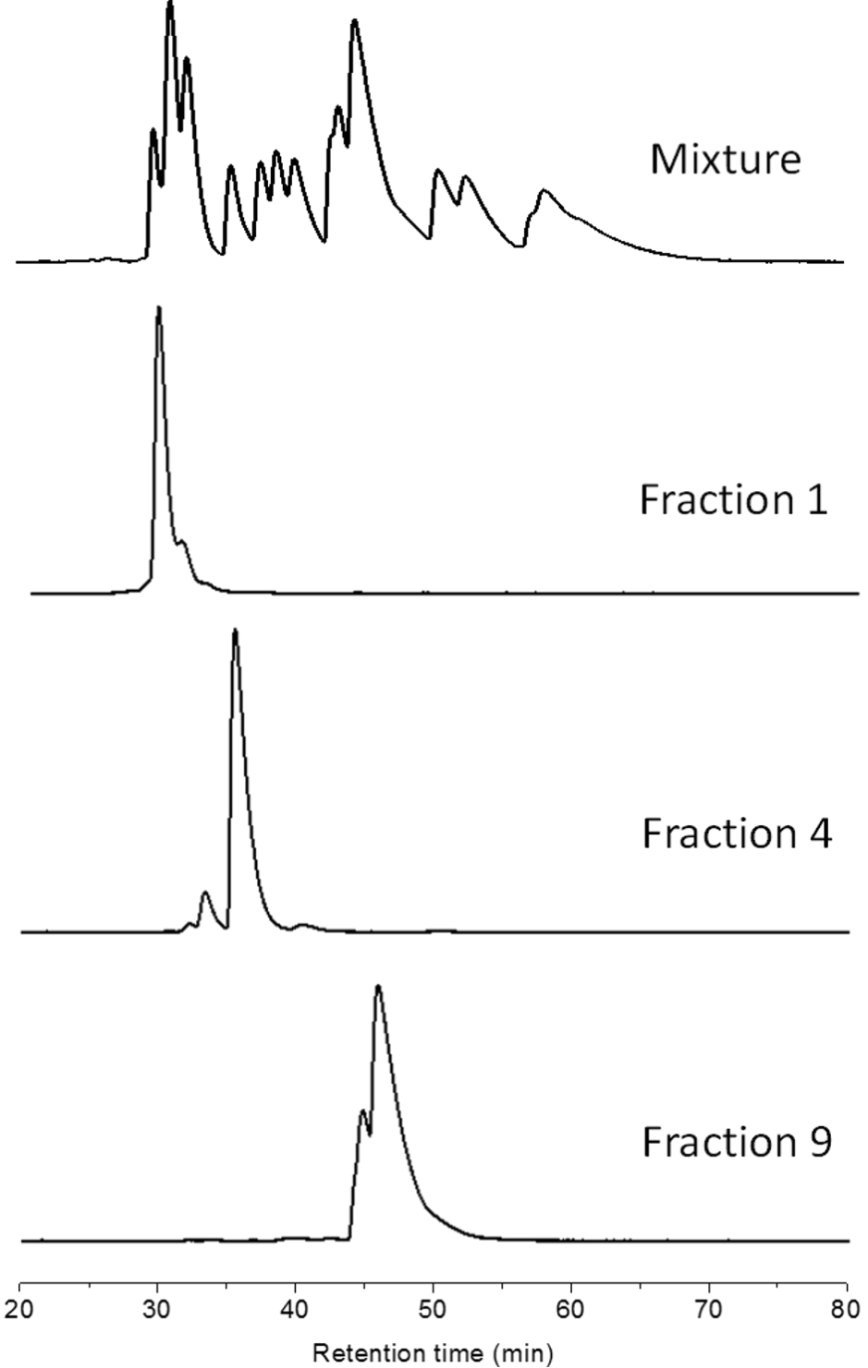
Chromatograms of IC_70_BA mixture and fractions 1, 4 and 9 separated by HPLC (Cosmosil Buckyprep-D column, toluene, 0.2 mL/min). Note that fraction 9 was further separated by flash chromatography (silica gel, toluene/cyclohexane 1:9) into fractions 9-1 and 9-2.

The synthesis of the isomeric mixture of IC_70_BA was achieved by heating C_70_ with indene at 180 °C in 1,2-dichlorobenzene [9]. Following the reaction, flash chromatography (silica gel, toluene: cyclohexane, 1:9) was performed to remove any excess reagents, mono-adducts of C_70_ as well as other impurities. In our previous work, the mixture of IC_70_BA was separated into two fractions by means of flash chromatography; however in this case these two fractions were combined and further purified by HPLC using a Cosmosil Buckyprep-D column (4.6 i.d. × 250 mm, toluene, 0.2 mL/min, UV detection 325 nm). More than thirteen peaks were observed in the HPLC chromatogram (Figure 3). From these, eleven fractions were collected by the liquid handler of the HPLC equipment (see details in Supporting Information File 1 and Figure S1). The cleanest fractions were 1, 4 and 9 as shown in the chromatograms (Figure 3) and fractions 2, 3, 9 and 11 contained the biggest portions of the original mixture, enough for device testing. Notably, fraction 9 contained two IC_70_BA species and was further separated by flash chromatography (silica gel, toluene/cyclohexane 1:9) into fraction 9-1 and fraction 9-2 which are known to contain the 2 o’clock-B isomer [9].

**Figure 4:**
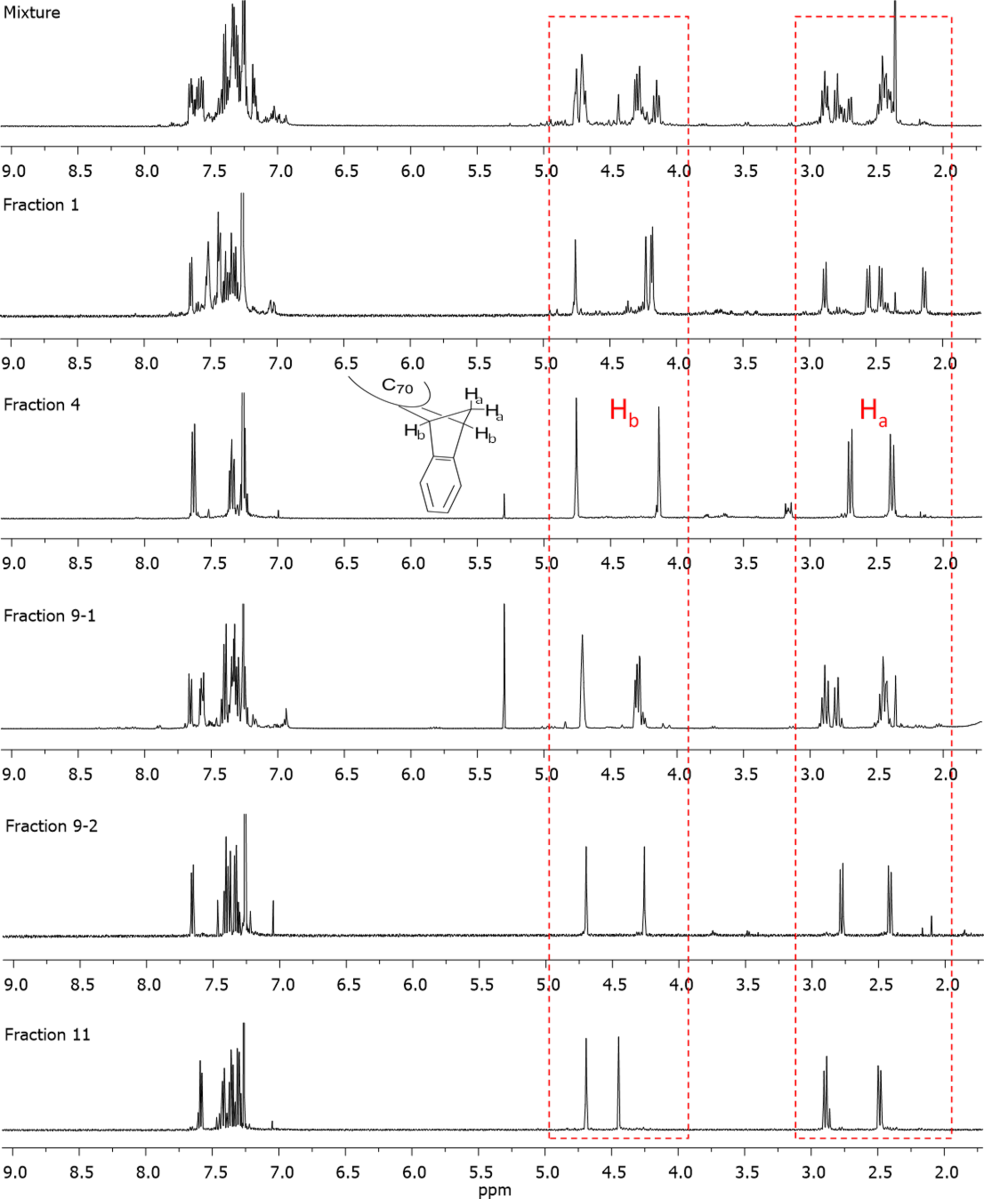
^1^H NMR spectrum of IC_70_BA fractions containing a major isomer species.

According to the ^1^H NMR spectra, there are five fractions which show clear identifiable proton resonances. These are fractions 1, 4, 9-1, 9-2 and 11 (Figure 4, see Supporting Information File 1, Figure S2 for comparisons between other fractions). The resonances located in the area of 4 ppm to 5 ppm belonged to CH protons (H_b_) of IC_70_BA, while the resonances between 2 ppm to 3 ppm were attributed to the CH_2_ protons (H_a_). Due to the limitations of the separating efficiency of HPLC, other fractions were either too low in yield or mixed with neighboring fractions. In those cases, the ^1^H NMR experiments did not provide useful information for identifying the configuration of isomers contained in the samples (see Supporting Information File 1).

As shown in Figure 2, some geometrical isomers of IC_70_BA belong to the *C*_2_ or *C_s_* point group while others are in the *C*_1_ point group. For isomers in *C*_2_ or *C_s_* configurations, each of their two substituents is in the same chemical environment. As a result, their –CH_2_ resonances should spilt into two doublet peaks and the –CH resonances should also spilt into two singlet or doublet peaks depending on whether it couples with the protons of the –CH_2_ group. According to the ^1^H NMR spectra, fractions 4, 9-2 and 11 were symmetrical isomers due to their simplified ^1^H resonances, while the compounds in fractions 1 and 9-1 did not have any plane or point of symmetry (Figure 4). Although fraction 3 included approximately 30% impurities of fraction 2, we could still observe clearly two sharp singlet resonances at 4.75 and 4.14 ppm, which suggested that the major compound in fraction 3 had *C*_2_ or *C_s_* symmetry (Supporting Information File 1, Figure S2). The ^13^C NMR spectrum of fractions 4, 9-2 and 11 showed 40 resonances in the 120–165 ppm region where the sp^2^ carbon resonances of the fullerene molecule were commonly observed (see Supporting Information File 1). This was a strong indication that these fractions contained *C*_2_ or *C_s_* symmetric derivatives. On the other hand, there were more than 70 resonances in that region for fractions 1 and 9-1. This was further evidence that the fullerene derivatives in fractions 1 and 9-1 did not have any plane or point of symmetry.

The separation mechanism of the Cosmosil Buckyprep-D column is based on the electronic π-orbital interactions between fullerene species and the nitrocarbazoyl-functionalized silica stationary phase [15]. As a consequence, it was envisaged that the fullerene derivatives with smaller π-conjugated area would interact less strongly with the stationary phase of the column and therefore elute faster than derivatives with larger π-surface. In addition, the substitution on fullerenes may block the interaction between fullerene and the stationary phase which also shortens the retention time on the Cosmosil Buckyprep-D column. Considering the configurations of the three major regioisomers of IC_70_BA, the 5 o’clock regioisomers are likely to elute first because the substituent geometry blocks the largest fullerene surface area. In contrast, the 12 o’clock regioisomers are expected to elute last as a result of the smallest angle between the two indene adducts. Applying logical deduction, we would anticipate that the isomers of IC_70_BA elute from the HPLC in the following order: 5 o’clock, 2 o’clock and then 12 o’clock. This is consistent with our previous isolation and identification of the known single isomer, 2 o’clock-B, which is located in the middle of the HPLC chromatogram (Supporting Information File 1, Figure S1 and Table S1) [9].

**Figure 5:**
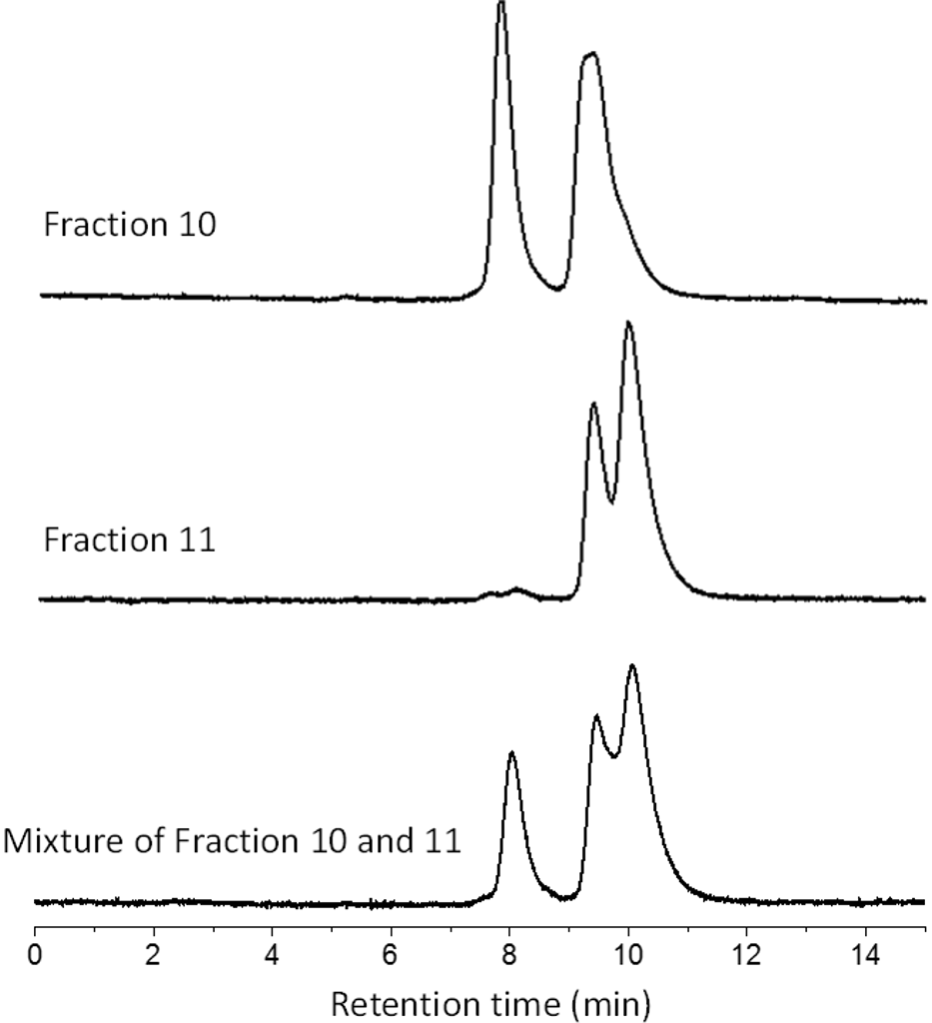
The retention time of the first species in fraction 10 is shorter than the species in fraction 11 on silica gel HPLC column (cyclohexane, 1.0 mL/min) providing information on the relative polarity of the compounds under investigation.

The fast HPLC elution time of fractions 1, 2 and 3 meant that these fractions were likely to contain 5 o’clock regioisomers (Supporting Information File 1, Figure S1 and Table S1). With symmetry information from NMR experiments, there is a high probability that fraction 1, assigned to the *C*_1_ point group, contained the 5 o’clock-A isomer (Figure 2b). Fraction 2 has a slightly shorter retention time relative to fraction 3. This indicates that fraction 2 could be in 5 o’clock-C configuration with its two indene substituents covering a larger conjugated area of C_70_ than fraction 3. Meanwhile, fractions 4 and 9-1 are more likely to be in the 2 o’clock-A and 2 o’clock-C configuration, respectively. That is because they are in the center of the HPLC chromatogram while in the *C*_1_ and *C*_2_ point groups, respectively. Fractions 10 and 11 are thought to be the 12 o’clock isomers, because of their locations in the rear of the HPLC stream. These two fractions were analyzed with a silica gel HPLC column (cyclohexane 1.0 mL/min), respectively, to assess their relative polarity. The HPLC chromatogram clearly illustrated that the retention time of fraction 10 was shorter than of fraction 11, which suggested that the configuration of fraction 10 was less polar than fraction 11 (Figure 5). With this information in mind, we are confident that fraction 10 contained the 12 o’clock-B isomer while fraction 11 contained the 12 o’clock-A isomer (Figure 2b).

**Figure 6:**
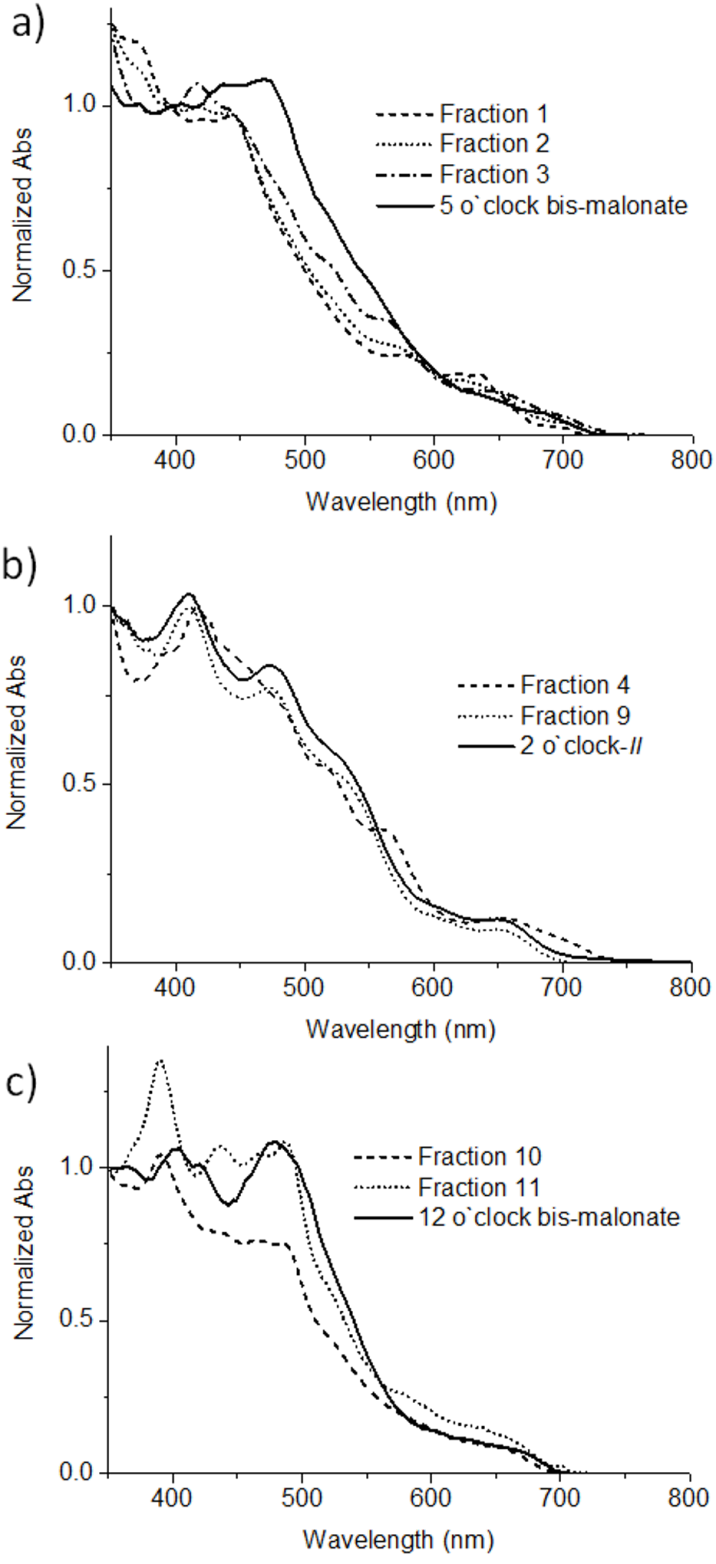
The UV–vis spectrum of each fraction of IC_70_BA as well as known C_70_ bisadducts: a) fraction 1, 2, 3 and 5 o`clock bis-malonate [17]; b) fraction 4, 9-1 and 2 o`clock-B of IC_70_BA [9]; c) fraction 10, 11 and 12 o`clock bis-malonate [17].

The assignments from chromatography and NMR experiments were supported by the analysis of the UV–vis absorption spectrum of the IC_70_BA fractions. It is widely known that the UV–vis spectrum of fullerene derivatives are highly correlated to their conjugated structures [16]. Therefore, comparison of the UV–vis spectrum of each fraction with known C_70_ bisadducts, for example the known 2 o’clock-B isomer of IC_70_BA [9] and C_70_ bis-malonate isomers [17], was expected to provide further information on adduct configurations. The UV–vis spectra of fractions 1, 2 and 3 showed very similar spectral features when compared with the spectrum of the 5 o’clock isomer of C_70_ bis-malonate, suggesting that they are all 5 o’clock isomers (Figure 6a). Similarly, the spectrum of fractions 4 and 9-1 matched that of the previously identified 2 o’clock-B IC_70_BA quite well. They all show an absorption maximum at 410 nm and a shoulder at around 478 nm (Figure 6b). Finally, fractions 10 and 11 showed a very similar UV–vis spectrum profile to the 12 o’clock C_70_ bis-malonate (Figure 6c). Thus the eight major regioisomers of IC_70_BA were identified. However, the remaining fractions 5–8, were also confirmed to be IC_70_BA isomers by mass spectrometry. Since the UV–vis spectrum of these fractions did not correlate to those of the known α-bonds C_70_ bisadducts (Supporting Information File 1, Figure S13), it was reasonable to expect that these fractions contained IC_70_BA compounds with at least one indene substitution located on non-α-bonds of C_70_. A summary of the isomer configuration assignments and related characterization data for the various fractions of IC_70_BA is shown in Table 1.

**Table 1:** Characterization data of various IC_70_BA fractions.

	Configuration of major isomer in the sample^a^	UV–vis **λ**_max_ (nm)^b^	Reduction *E*_½_ (eV)^c^	*E*_LUMO_ (eV)^d^

IC_70_BA mixture	–	–	−1.24	−3.56
Fraction 1	5 o’clock-A	447 (1.1)	−1.13	−3.67
Fraction 2^e^	5 o’clock-C	446 (2.1)	−1.19	−3.61
Fraction 3	5 o’clock-B	417 (1.8)	−1.19	−3.61
Fraction 4	2 o’clock-C	417 (1.6)	−1.22	−3.58
Fraction 9-1	2 o’clock-A	410 (1.2)	−1.15	−3.65
Fraction 9-2	2 o’clock-B	411 (2.1)	−1.25	−3.55
Fraction 10^e^	12 o’clock-B	390 (1.2)	−1.20	−3.60
Fraction 11	12 o’clock-A	389 (2.0)	−1.17	−3.63

^a^Assignments made using a combination of NMR, UV–vis and chromatographic experiment data; ^b^Solution UV–vis data. Absorption coefficient (×10^3^ M^−1^ cm^−1^) in brackets; ^c^Half-wave potential of first reduction; ^d^Calculated from *E*_LUMO_ = −4.8 + *E*_½_; ^e^Fractions 2 and 10 contained a substantial quantity of other IC_70_BA isomers.

In order to analyze the electrochemical properties of the IC_70_BA fractions, cyclic voltammetry was performed on each fraction. The first reduction potentials of all 11 fractions were found to be in the range of −1.13 to −1.25 eV versus that of ferrocene/ferrocenium (see Supporting Information File 1, Figure S14). Therefore, the LUMO energy level of all single isomer of IC_70_BA and the isomer mixture were close to −3.6 eV. The UV–vis and electrochemical characterization are summarized in Table 1.

**Figure 7:**
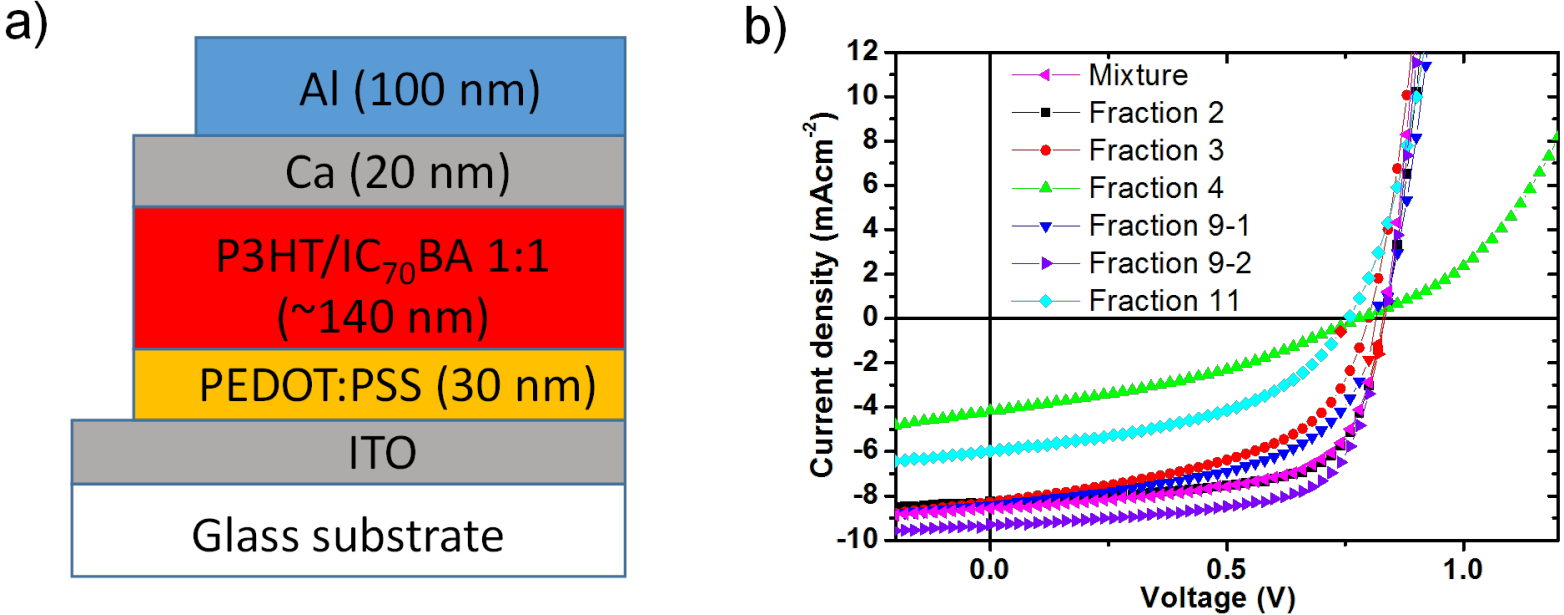
Schematic diagram of the architecture of BHJ solar cell devices (a) and *J*−*V* curves of the devices containing P3HT and each IC_70_BA fractions (b).

The solar cell devices were fabricated in the architecture: ITO/PEDOT:PSS/active layer/Ca/Al (Figure 7a). The active layer consisted of a blend of the listed fractions of IC_70_BA with P3HT (see details in Table 2), in the ratio of 1:1 by weight. The fractions were chosen depending on their abundance and purities (Supporting Information File 1, Table S1). The open circuit volatge (*V*_oc_) of all devices were at around 0.8 eV which corresponded to the *E*_LUMO_ values from electrochemical experiments (Table 1 and Table 2). Figure 7b shows the current density–voltage (*J*–*V*) curves of the solar cell devices, under the illumination of AM1.5G, 100 mW cm^−2^. The photovoltaic performance data of the devices are summarized in Table 2 for a clear comparison between various ICBA fractions. The device with fraction 9-2 exhibits highest power conversion efficiencies (PCE) of 5.2% which is superior than the ICBA mixture (PCE of 4.5%) based device. The enhanced performance might be due to the favorable molecular packing in the active layer due to crystalline isomer fraction 9-1 as well as nanoscale phase separation morphology (Supporting Information File 1, Figure S16d) of active layer blend [14]. The other fullerene fractions (2, 3 and 9-1) showed the moderate device performance with short circuit current density (*J*_sc_) and PCE value in the range of 8.0–8.3 mA/cm^2^ and 3.1–4.4%, respectively. However, the *J*_sc_ and PCE and of the devices containing fractions 4 and 11 were significantly lower in performance compared (Table 2) to the devices of other fractions.

**Table 2:** Performance of BHJ solar cell devices based on a blend of P3HT and each IC_70_BA fractions as the active layer.^a^

Active layer P3HT/IC_70_BA (1:1)	*J*_sc_ (mA/cm^2^)	*V*_oc_ (V)	FF (%)	PCE (%)

Mixture	8.6 ± 0.30	0.82 ± 0.02	64 ± 3	4.5 ± 0.25
Fraction 2	8.1 ± 0.20	0.80 ± 0.02	66 ± 2	4.4 ± 0.20
Fraction 3	8.0 ± 0.30	0.76 ± 0.02	50 ± 3	3.1 ± 0.30
Fraction 4	3.9 ± 0.35	0.74 ± 0.02	34 ± 4	0.9 ± 0.40
Fraction 9-1	8.2 ± 0.25	0.78 ± 0.02	55 ± 2	3.6 ± 0.25
Fraction 9-2	9.3 ± 0.15	0.82 ± 0.02	68 ± 2	5.2 ± 0.15
Fraction 11	5.7 ± 0.30	0.72 ± 0.02	44 ± 3	1.8 ± 0.30

^a^The data shown are the average values obtained from 10 devices with standard deviation.

It is important to point out that fraction 4 had the highest purity among all the fractions (Supporting Information File 1, Table S1), which demonstrated that the purity is not the only factor influencing the performance of devices. Taking into account both fraction purity and assigned geometric configuration, a surprising trend emerged with apparent decrease in *J*_sc_ with increasing fraction purity and crystallinity. The crystallinity of a given fraction can be considered as dependent on the symmetry of the assigned isomer configuration. That is, the degree of symmetry varies in the order *C*_1_ < *C*_2_ < *C_s_*, which resulted in the order of crystallinity for the five fractions ranked as 9 < 2 ≈ 3 < 4 < 11. Considering fraction 4 had the highest purity, the order of crystallinity may be modified to 9 < 2 ≈ 3 < 11 < 4, which corresponded to the decreasing *J*_sc_ of the devices containing these fractions. One way to rationalise this observation is that increasing crystallinity of IC_70_BA can improve the charge carrier mobility of the bulk material but the miscibility with the P3HT electron donor material can also change. A key feature of efficient BHJ solar cell devices is the nanoscale phase separation of the electron donor and acceptor materials into continuous interpenetrating networks. Some indications on the degree of phase separation can be obtained in tapping mode atomic force microscopy (AFM) experiments (see Supporting Information File 1 for experimental details). Both height and phase AFM images suggested unfavourable phase separation for blend films containing IC_70_BA fractions 4 and 11 with domain sizes in the micrometre range (Supporting Information File 1, Figure S16c and S16e). This larger domain size is usually detrimental for charge separation and this is reflected in the lower *J*_sc_ and PCE for the devices containing fractions 4 and 11 as shown in Table 2.

## Conclusion

Herein we report the successful isolation of isomers of IC_70_BA through HPLC. Eleven distinct fractions were collected and analyzed to identify the various geometrical and regioisomers of this fullerene derivative. Furthermore, photophysical and electrochemical characterization was performed to evaluate the properties of these materials. From the eleven fractions, all major (α-bond) regioisomers of IC_70_BAs were identified with the details of their configuration and symmetry factors confirmed. It was found that material purity and crystallinity and their effects on the thin film nanostructure are key factors in determining the performance of these fullerene derivatives in BHJ solar cell devices. With the observations in this study, it can be argued that the success of IC_70_BA (and in extension, IC_60_BA) as the electron acceptor component in BHJ solar cells is serendipitous and surprising given the large number of chemical structures involved. It is noteworthy that there have been several studies on reducing the number of isomers in fullerene bisadduct materials using synthetic strategies and about the successful application in solar cell devices [18–22]. While high material purity and composition is generally considered an advantage for organic electronic materials, the material criteria for bulk heterojunction organic solar cell applications is less clear. This is owing to the fact that bulk heterojunctions are a mixture of at least two materials (an electron donor and an electron acceptor) with the film nanostructure being extremely important for the device performance.

## Supporting Information

File 1 Details on the separation procedure and characterization of the materials as well as device fabrication and testing.
