# Supplementary material for: Separation and identification of indene–C70 bisadduct isomers
Source: Beilstein J Org Chem. 2016 May 6;12:903–11. doi: 10.3762/bjoc.12.88 (PMC4901934; doi:10.3762/bjoc.12.88)
Supplement: File 1 — Details on the separation procedure and characterization of the materials as well as device fabrication and testing. [file Beilstein_J_Org_Chem-12-903-s001.pdf]

Supporting Information

for

Separation and identification of indene–C_{70} bisadduct isomers

Bolong Zhang, Jegadesan Subbiah, David J. Jones and Wallace W. H. Wong*

Address: School of Chemistry, Bio21 Institute, University of Melbourne, 30 Flemington Rd, Parkville, Victoria, 3010, Australia.

Email: Wallace W. H. Wong - wwhwong@unimelb.edu.au

*Corresponding author

Details on the separation procedure and characterization of the materials as well as device fabrication and testing

Table of contents

General experimental details s2
Experimental section s2
NMR spectrums of each fractions........ s6
UV–vis spectrum of $IC_{70}BA$ fractions 5 to 8 ....s11
Electrochemical properties s12
Device fabrication and characterization........ s13

General experimental details

C_{70} (BuckyUSA, 98%), indene (Sigma-Aldrich), *o*-dichlorobenzene (Sigma-Aldrich) and HPLC degree toluene (Merck) were used in the experiment as received from suppliers. Poly(3-hexylthiophene) was purchased from Merck KGaA (lisicon® SP001).

Flash chromatography purification was performed following standard chromatographic methods on silica gel (Merck Silica Gel 60, 0.040-0.063 mm, 230-400 mesh ASTM). Analytical HPLC was carried out using a standard HPLC system with a UV–vis detector. A Cosmosil® Buckyprep-D column (4.6 ID × 250 mm) was used with toluene as eluent (1 mL/min) and UV detection was recorded at 325 nm.

^{1}H NMR spectroscopy was performed using the Varian Inova-400 (400 MHz). ^{13}C NMR spectroscopy was performed using Oxford 500. FT-IR spectra were obtained using a Perkin Elmer Spectrum One FT-IR spectrometer while UV-vis spectra were recorded using a Cary UV–vis spectrometer. All high resolution mass spectrometry (HRMS) experiments were conducted with use of a commercially available hybrid linear ion trap and Fourier-transform ion cyclotron resonance mass spectrometer, equipped with electrospray ionisation (ESI). Cyclic voltammetry (CV) measurements were carried out using a Solartron 1287A Potentiostat/Galvanostat.

Experimental section

The crude $IC_{70}BA$ was synthesized as reported previously.[1] Fullerene C_{70} (1 g, 1.19 mmol) was dissolved in *o*-dichlorobenzene (50 mL) at 180 °C under nitrogen atmosphere. Indene was injected to the system one equivalent at a time (0.14 g, 1.19 mmol) and around 10 equivalents were added over the course of 10 hours. The reaction was monitored regularly by thin layer chromatography (SiO_2, cyclohexane/toluene 9:1).

Flash chromatography (SiO_2, cyclohexane/toluene 9:1) was performed on the the crude product to remove C_{70}, monoadducts, multiaddcts and other impurities. The mixture of $IC_{70}BAs$ then was separated by HPLC (Buckyprep-D, HPLC degree toluene, 0.2 ml/min, 325 nm wavelength for UV–vis detector). The liquid handler of HPLC injected the eluent flow into 60 tubes automatically from the 26th minute to 56th minute of the retention time. Then the solution in each tubes was collected together based on the chromatogram to give out the 11 fractions (Figure S1 and Table S1).

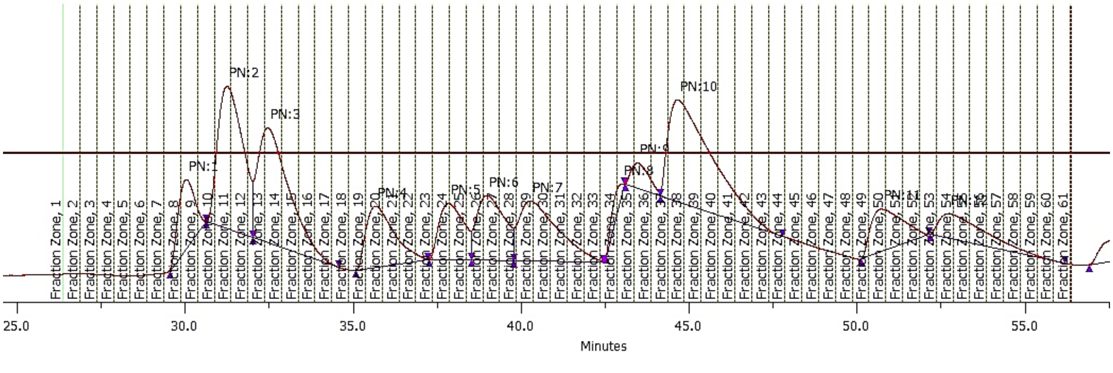

Figure S1: The eleunt flow was separated into 60 tubes.

Table S1: The ingredient of each fractions.

Fractions	Tubes	Proportion of product mixture %	Integration of major peak %
Fraction 1	7, 8	4	81
Fraction 2	10, 11	12	71
Fraction 3	12, 13, 14, 15, 16	11	61
Fraction 4	18, 19, 20, 21, 22	5	89
Fraction 5	23, 24	4	63
Fraction 6	25, 26, 27	5	60
Fraction 7	28, 29, 30, 31, 32	7	67
Fraction 8	33, 34, 35	9	38
Fraction 9	37, 38, 39, 40, 41, 42, 43, 44	27	82
Fraction 10	48, 49, 50, 51	6	32
Fraction 11	53, 54, 55, 56, 57, 58	10	73

Fraction 1: ^{1}H NMR (400 MHz, $CDCl_3$) δ = 7.63 (d, *J*=7.3, 1H), 7.53 – 7.47 (m, 2H), 7.44 – 7.40 (m, 2H), 7.38 – 7.28 (m, 3H), 4.74 (s, 1H), 4.21 (s, 1H), 4.17 (d, *J*=5.3, 2H), 2.87 (d, *J*=10.0, 1H), 2.54 (d, *J*=10.2, 1H), 2.45 (d, *J*=10.0, 1H), 2.12 (d, *J*=10.1, 1H). ^{13}C NMR (126 MHz, $CDCl_3$) δ = 161.05, 160.63, 156.64, 156.19, 154.76, 154.41, 154.06, 153.95, 153.87, 152.67, 152.42, 152.08, 151.92, 151.87, 151.57, 150.24, 150.20, 149.93, 149.75, 149.59, 149.46, 149.40, 149.29, 148.67, 148.64, 148.40, 148.35, 148.19, 147.78, 147.57, 147.13, 147.09, 147.04, 146.96, 146.54, 145.73, 145.64, 145.61, 145.58, 145.39, 145.25, 145.09, 145.00, 144.19, 144.14, 142.99, 142.92, 142.35, 142.22, 139.48, 139.31, 139.05, 138.61, 138.46, 138.36, 134.25, 133.50, 132.97, 132.21, 132.03, 131.94, 127.73, 127.22, 127.15, 123.90, 123.72, 123.57, 69.28, 67.59, 65.55, 65.52, 58.19, 57.75, 57.27, 54.96, 46.18, 45.65, 29.69. FT-IR (cm^{-1}): 3750.3, 2923.1, 1699.9, 1559.0, 1441.0, 1262.9, 749.1, 662.1; HRMS ESI+ (m/z): calcd. for $C_{88}H_{16}$, 1072.1252; found M^+, 1072.1271.

Fraction 2: ^{1}H NMR (400 MHz, $CDCl_3$) δ = 7.63 (d, *J*=7.3, 1H), 7.58 (d, *J*=7.3, 1H), 7.53 – 7.48 (m, 1H), 7.43 – 7.40 (m, 1H), 7.38 – 7.27 (m, 4H), 4.76 (s, 2H), 4.16 (s, 2H), 2.94 – 2.82 (m, 2H), 2.40 (dt, *J*=10.0, 1.7, 2H). FT-IR (cm^{-1}): 2923.4, 1716.8, 1440.9, 1262.6, 1212.2, 1008.0, 948.2, 748.9, 659.4; HRMS ESI+ (m/z): calcd. for $C_{88}H_{16}$, 1072.1252; found M^+, 1072.1270.

Fraction 3: ^{1}H NMR (400 MHz, $CDCl_3$) δ = 7.63 (d, *J*=7.3, 1H), 7.59 (dd, *J*=7.4, 3.6, 2H), 7.52 (ddd, *J*=13.7, 8.8, 5.1, 1H), 7.39 – 7.28 (m, 4H), 4.75 (dt, *J*=3.3, 1.5, 2H), 4.15 (dd, *J*=8.7, 1.5, 2H), 2.76 – 2.66 (m, 2H), 2.42 – 2.35 (m, 2H). ^{13}C NMR (126 MHz, $CDCl_3$) δ = 160.69, 160.29, 157.02, 156.91, 156.62, 156.55, 156.46, 156.12, 154.63, 154.51, 154.49, 154.44, 154.23, 154.10, 153.53, 153.47, 153.16, 152.90, 152.41, 152.37, 152.34, 151.95, 151.65, 151.58, 151.44, 151.43, 151.35, 151.32, 151.28, 151.08, 151.04, 150.95, 150.64, 150.53, 150.50, 150.44, 150.41, 150.37, 150.31, 150.25, 150.12, 150.02, 149.99, 149.85, 149.69, 149.65, 149.63, 149.60, 149.59, 149.53, 149.21, 149.08, 148.73, 148.70, 148.63, 148.60, 148.56, 148.44, 148.42, 148.32, 148.30, 148.29, 148.15, 148.07, 148.04, 147.91, 147.81, 147.64, 147.58, 147.55, 147.48, 147.35, 147.25, 147.22, 147.21, 147.15, 147.11, 147.05, 146.98, 146.93, 146.85, 146.45, 146.24, 146.05, 146.01, 145.64, 145.58, 145.40, 145.36, 145.29, 145.23, 145.21, 145.17, 145.03, 144.92, 144.87, 144.69,

144.30, 144.25, 143.51, 143.48, 143.16, 143.12, 142.87, 142.84, 142.78, 142.72, 142.66, 142.64, 142.60, 142.46, 142.44, 142.40, 142.37, 142.36, 142.10, 141.97, 141.84, 139.93, 139.51, 139.42, 139.38, 139.18, 139.16, 138.71, 137.96, 137.86, 137.62, 137.54, 136.71, 136.61, 136.50, 134.33, 134.16, 133.83, 133.80, 133.68, 133.53, 133.44, 133.41, 133.37, 133.29, 133.23, 133.13, 133.00, 132.16, 131.87, 131.71, 131.54, 131.45, 131.13, 131.08, 129.02, 127.24, 127.21, 127.19, 127.16, 127.13, 127.09, 126.89, 126.87, 126.80, 124.02, 124.00, 123.96, 123.91, 123.88, 123.82, 123.72, 123.69, 123.58, 123.53, 123.51, 123.43, 69.29, 68.75, 68.71, 68.68, 67.56, 67.20, 67.18, 67.14, 66.36, 66.24, 65.90, 65.33, 65.30, 58.53, 58.38, 58.27, 58.25, 58.23, 58.19, 58.10, 57.98, 57.76, 57.28, 55.88, 55.83, 55.61, 54.99, 47.38, 46.92, 46.15, 45.79, 45.74, 45.53. FT-IR (cm^{-1}): 2923.3, 1716.8, 1442.7, 1262.9, 1214.3, 1022.0, 795.2, 752.3, 660.5; HRMS ESI+ (m/z): calcd. for $C_{88}H_{16}$, 1072.1252; found M^+, 1072.1270.

Fraction 4: ^{1}H NMR (400 MHz, $CDCl_3$) δ = 7.61 (q, *J*=9.4, 8.1, 3H), 7.39 – 7.30 (m, 3H), 7.30 – 7.28 (m, 1H), 7.26 (d, *J*=1.8, 1H), 4.74 (t, *J*=2.1, 2H), 4.13 (dd, *J*=7.7, 1.6, 2H), 2.81 – 2.63 (m, 2H), 2.41 – 2.35 (m, 2H). ^{13}C NMR (126 MHz, $CDCl_3$) δ = 156.61, 156.58, 154.38, 152.29, 151.72, 151.15, 151.05, 150.89, 150.47, 150.25, 150.05, 149.38, 148.70, 148.58, 148.04, 147.30, 147.08, 146.91, 146.08, 145.37, 144.85, 143.18, 142.95, 142.80, 142.62, 142.29, 141.82, 140.01, 137.86, 136.40, 134.35, 133.40, 133.25, 133.08, 131.40, 127.08, 123.99, 123.76, 68.64, 67.15, 58.18, 55.56, 45.43. FT-IR (cm^{-1}): 3749.8, 2921.0, 1716.5, 1542.0, 1443.7, 1410.5, 1264.2, 1217.7, 1073.3, 1015.6, 946.1, 751.4, 677.7, 659.8; HRMS ESI+ (m/z): calcd. for $C_{88}H_{16}$, 1072.1252; found M^+, 1072.1273.

Fraction 5: ^{1}H NMR (400 MHz, $CDCl_3$) δ = 7.57 – 7.41 (m, 4H), 7.38 – 7.28 (m, 4H), 4.28 (d, *J*=14.3, 4H), 3.00 (dd, *J*=25.3, 9.9, 2H), 2.53 (d, *J*=10.0, 2H). FT-IR (cm^{-1}): 2923.0, 1716.5, 1443.3, 1262.8, 1215.4, 1032.1, 947.0, 751.4, 661.0; HRMS ESI+ (m/z): calcd. for $C_{88}H_{16}$, 1072.1252; found M^+, 1072.1270.

Fraction 6: ^{1}H NMR (400 MHz, $CDCl_3$) δ = 7.61 (t, *J*=7.1, 1H), 7.57 – 7.52 (m, 1H), 7.52 – 7.48 (m, 1H), 7.46 – 7.42 (m, 1H), 7.37 – 7.28 (m, 3H), 7.27 – 7.25 (m, 1H), 4.87 (d, *J*=19.6, 1H), 4.28 (d, *J*=13.6, 2H), 4.12 (s, 1H), 3.09 – 2.86 (m, 2H), 2.57 – 2.40 (m, 2H). FT-IR (cm^{-1}): 2967.6, 1716.5, 1450.8, 1263.1, 1214.4, 1112.6, 1034.3, 948.3, 752.1, 661.1; HRMS ESI+ (m/z): calcd. for $C_{88}H_{16}$, 1072.1252; found M^+, 1072.1262.

Fraction 7: ^{1}H NMR (400 MHz, $CDCl_3$) δ = 7.73 (d, *J*=7.3, 1H), 7.60 (d, *J*=7.2, 1H), 7.56 – 7.47 (m, 2H), 7.44 – 7.28 (m, 4H), 4.88 – 4.82 (m, 1H), 4.34 – 4.07 (m, 3H), 3.02 – 2.84 (m, 2H), 2.53 – 2.36 (m, 2H). FT-IR (cm^{-1}): 2923.2, 1716.5, 1428.4, 1261.8, 1213.6, 1086.6, 1036.7, 947.3, 794.3, 751.3, 656.8; HRMS ESI+ (m/z): calcd. for $C_{88}H_{16}$, 1072.1252; found M^+, 1072.1258.

Fraction 8: ^{1}H NMR (400 MHz, $CDCl_3$) δ = 7.67 (d, *J*=7.3, 1H), 7.56 (d, *J*=7.1, 1H), 7.53 – 7.36 (m, 2H), 7.36 – 7.26 (m, 4H), 4.85 – 4.67 (m, 1H), 4.33 – 4.25 (m, 2H), 4.22 (d, *J*=1.7, 1H), 2.96 – 2.82 (m, 2H), 2.52 – 2.38 (m, 2H). FT-IR (cm^{-1}): 2923.2, 285.0, 1716.6, 1440.3, 1262.7, 1213.2, 1029.8, 948.6, 794.1, 750.9, 667.3; HRMS ESI+ (m/z): calcd. for $C_{88}H_{16}$, 1072.1252; found M^+, 1072.1267.

Fraction 9-1: ^{1}H NMR (400 MHz, $CDCl_3$) δ = 7.69 – 7.61 (m, 1H), 7.59 – 7.52 (m, 1H), 7.42 – 7.26 (m, 6H), 4.69 (ddd, *J*=6.9, 3.5, 1.6, 2H), 4.33 – 4.21 (m, 2H), 2.95 – 2.71 (m, 2H), 2.51 – 2.37 (m, 2H). ^{13}C NMR (126 MHz, $CDCl_3$) δ = 162.02, 161.99, 157.73, 157.64, 157.44, 157.09, 156.66, 156.61, 155.98, 155.92, 155.70, 155.68, 155.34, 155.21, 152.13, 151.78, 151.74, 151.72, 151.68, 151.66, 151.62, 151.51, 151.32, 151.30, 151.25, 151.08, 150.56, 150.36, 150.27, 150.24, 150.18, 150.14, 149.26, 149.21, 149.16, 149.10, 149.06, 149.01, 148.98, 148.73, 148.67, 148.51, 148.37, 148.36, 148.15, 148.01, 147.88, 147.68, 147.66,

147.60, 147.54, 146.95, 146.66, 146.12, 145.45, 145.36, 145.33, 145.17, 145.15, 145.13, 144.24, 144.23, 144.11, 144.09, 144.04, 143.64, 143.43, 143.39, 143.26, 142.83, 142.79, 142.74, 141.39, 141.36, 141.34, 141.15, 141.13, 141.08, 140.74, 140.60, 140.24, 140.17, 140.01, 139.03, 138.94, 138.81, 138.76, 138.38, 138.30, 137.09, 136.59, 136.56, 134.28, 134.23, 133.89, 133.60, 133.42, 132.75, 132.45, 132.28, 132.00, 131.83, 131.79, 129.02, 128.20, 127.23, 127.21, 127.20, 127.17, 127.13, 127.11, 127.03, 124.10, 123.99, 123.96, 123.90, 123.89, 123.84, 123.69, 123.67, 123.63, 123.59, 123.45, 69.00, 68.96, 68.94, 67.34, 67.30, 58.38, 58.00, 57.94, 57.89, 55.40, 55.32, 55.19, 55.12, 46.33, 46.32, 46.13, 45.84, 31.92, 30.15, 29.70, 29.65, 29.36, 22.69, 14.12. FT-IR (cm^{-1}): 2921.2, 2851.4, 1701.0, 1461.5, 1377.0, 1262.8, 1030.5, 795.0, 751.2, 669.9; HRMS ESI+ (m/z): calcd. for $C_{88}H_{16}$, 1072.1252; found M^+, 1072.1263.

Fraction 10: ^{1}H NMR (400 MHz, $CDCl_3$) δ = 7.64 (d, *J*=7.5, 1H), 7.57 (t, *J*=6.5, 1H), 7.47 – 7.42 (m, 2H), 7.40 – 7.28 (m, 4H), 4.68 (d, *J*=8.6, 2H), 4.23 (d, *J*=3.7, 2H), 2.51 – 2.39 (m, 4H). FT-IR (cm^{-1}): 2922.6, 1716.6, 1699.9, 1542.1, 1457.1, 1413.4, 1263.4, 1218.3, 1026.8, 771.2, 753.0, 665.5; HRMS ESI+ (m/z): calcd. for $C_{88}H_{16}$, 1072.1252; found M^+, 1072.1273.

Fraction 11: ^{1}H NMR (400 MHz, $CDCl_3$) δ = 7.60 – 7.55 (m, 2H), 7.44 – 7.37 (m, 2H), 7.37 – 7.30 (m, 2H), 7.29 (ddt, *J*=6.8, 1.2, 0.5, 2H), 4.67 (d, *J*=1.6, 2H), 4.46 – 4.39 (m, 2H), 2.87 (dt, *J*=9.9, 1.5, 2H), 2.52 – 2.41 (m, 2H). ^{13}C NMR (126 MHz, $CDCl_3$) δ = 157.46, 157.16, 155.36, 154.87, 152.34, 152.00, 151.94, 151.76, 151.27, 151.15, 151.11, 150.74, 150.51, 149.31, 148.91, 147.76, 147.60, 145.35, 145.27, 141.79, 141.56, 141.41, 141.37, 141.35, 141.23, 141.08, 140.53, 137.50, 137.16, 136.42, 133.41, 132.83, 132.17, 131.82, 131.40, 131.34, 127.22, 127.15, 123.97, 123.95, 123.72, 76.75, 68.20, 67.42, 58.04, 55.91, 46.36, 29.70. FT-IR (cm^{-1}): 2974.2, 1699.8, 1574.5, 1442.8, 1413.7, 1214.9, 1152.1, 1027.0, 795.6, 750.0, 665.8; HRMS ESI+ (m/z): calcd. for $C_{88}H_{16}$, 1072.1252; found M^+, 1072.1270.

NMR data of $IC_{70}BA$ fractions

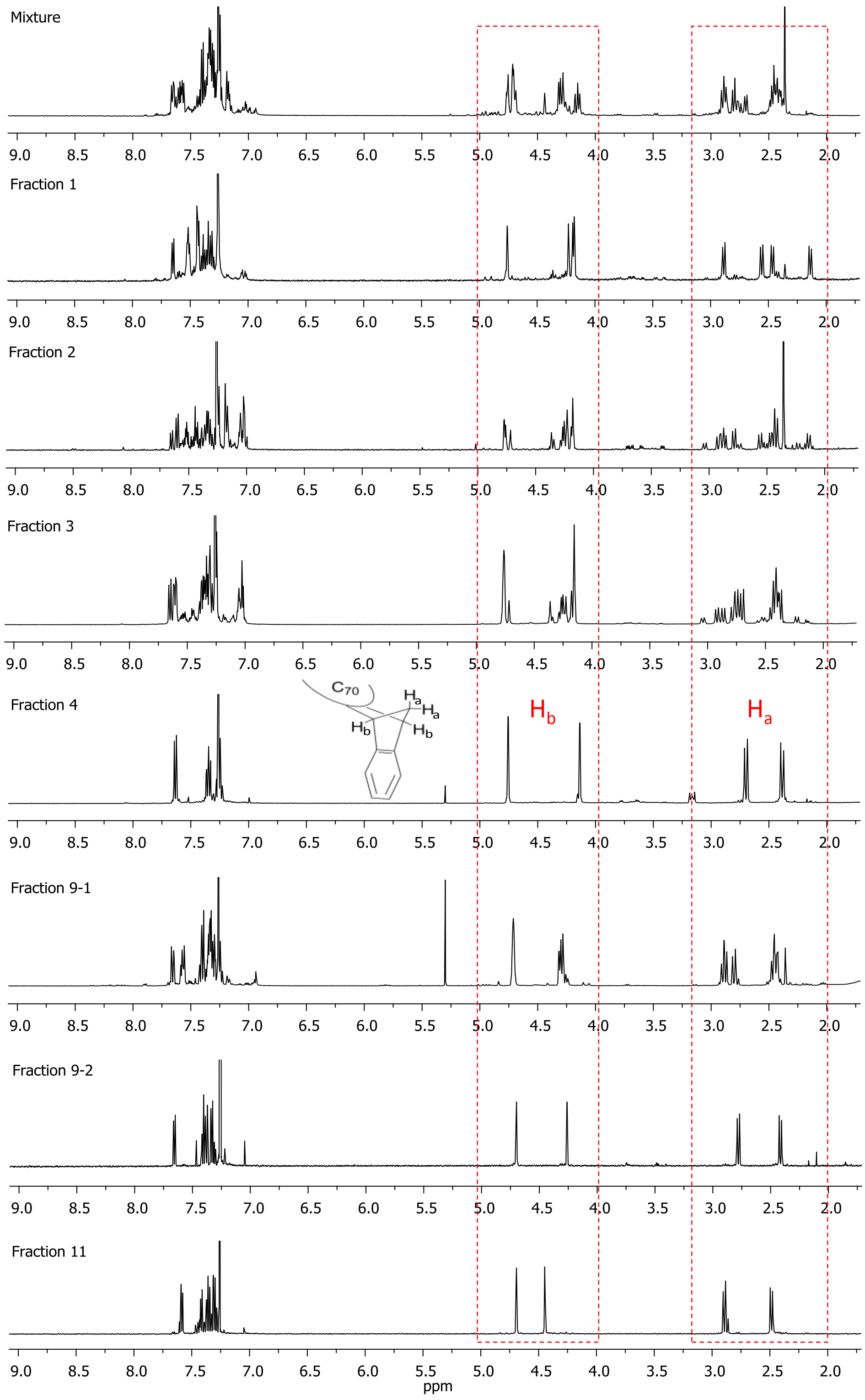

Figure S2: A comparison of ^1H NMR spectrum of $IC_{70}BA$ fractions.

^{1}H and ^{13}C NMR Spectrum of Individual $IC_{70}BA$ Fractions

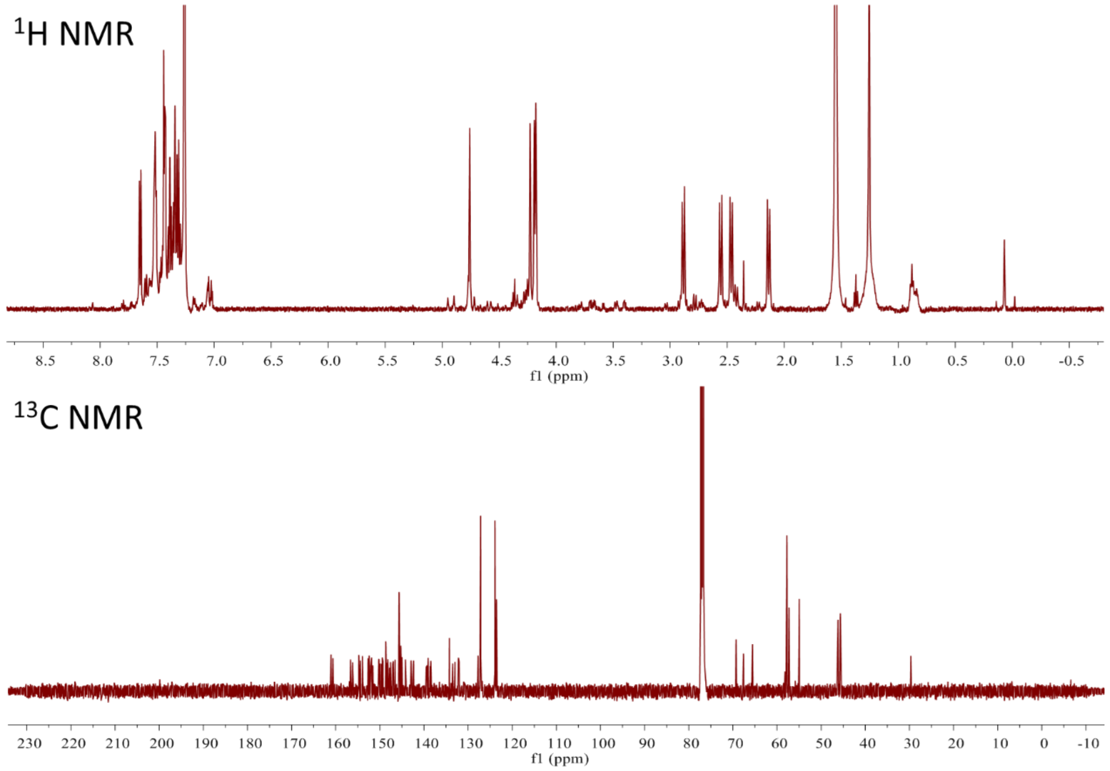

Figure S3. ^{1}H and ^{13}C NMR spectrum of Fraction 1.

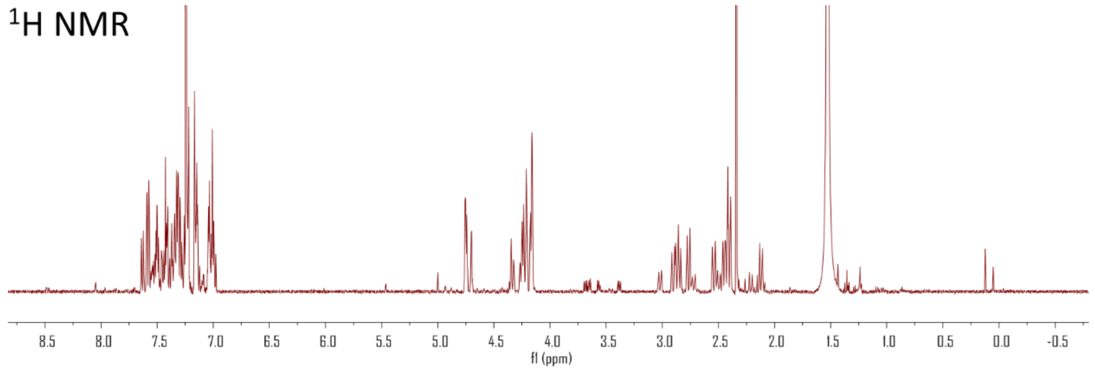

Figure S4: ^{1}H NMR spectrum of Fraction 2.

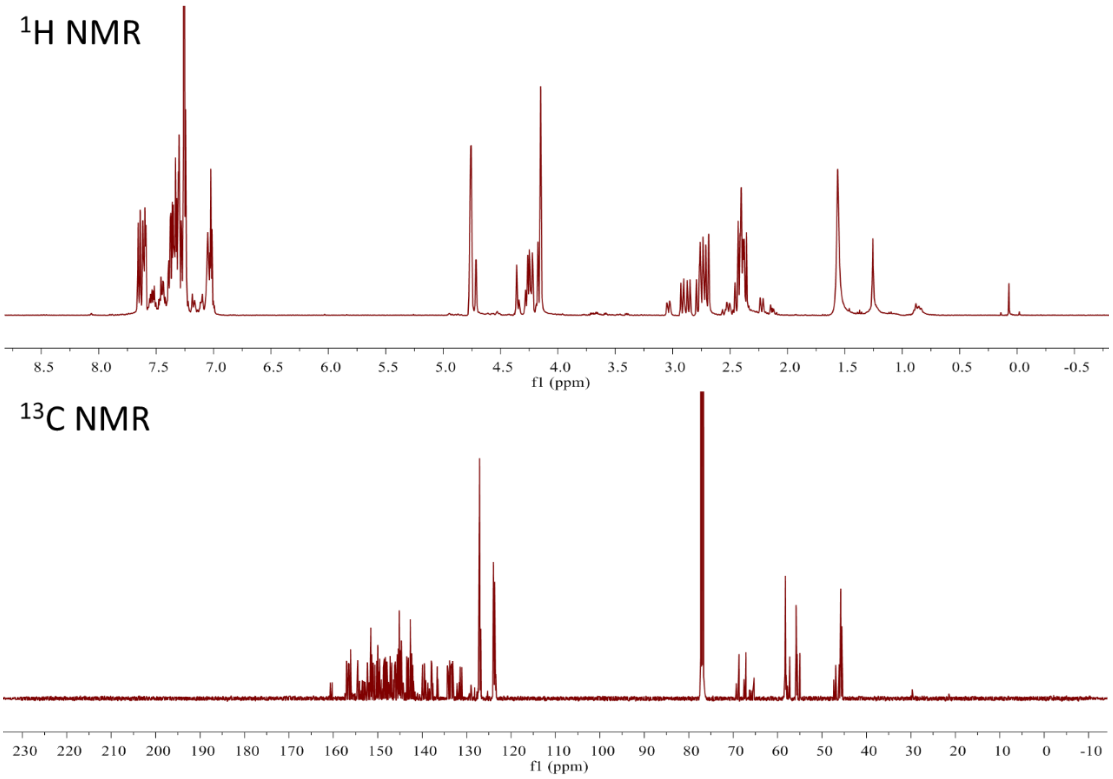

Figure S5: ^{1}H and ^{13}C NMR spectrum of Fraction 3.

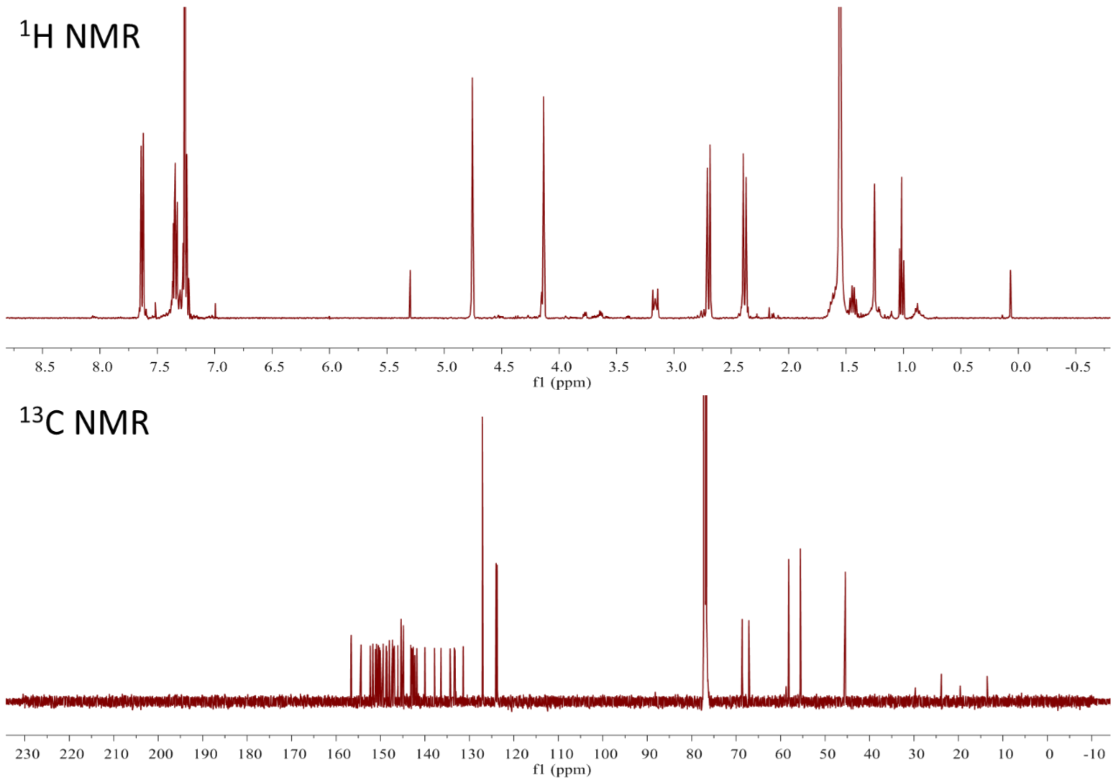

Figure S6: ^{1}H and ^{13}C NMR spectrum of Fraction 4.

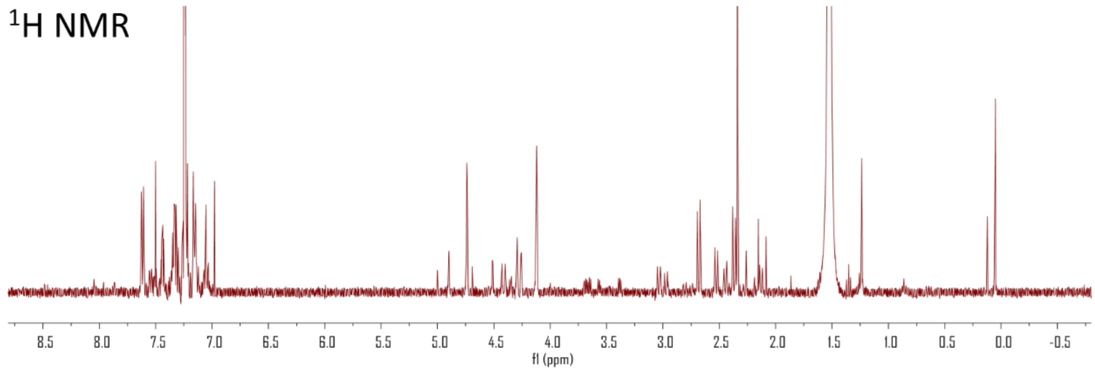

Figure S7: ¹H NMR spectrum of Fraction 5.

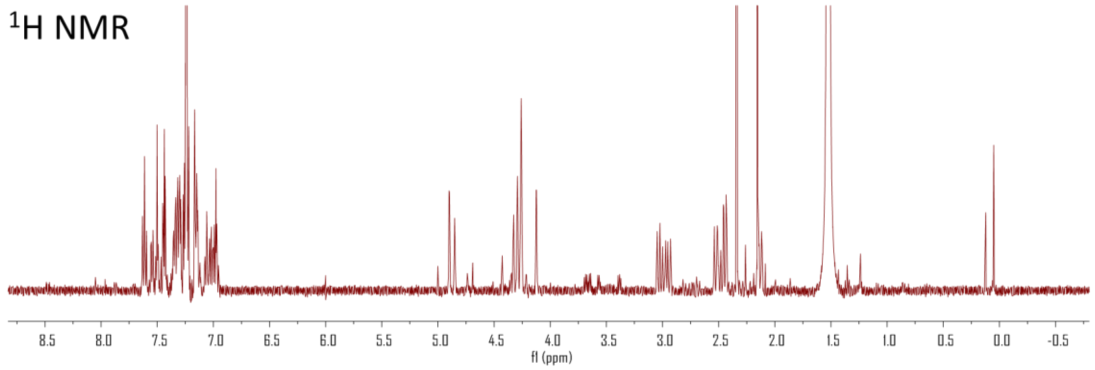

Figure S8: ¹H NMR spectrum of Fraction 6.

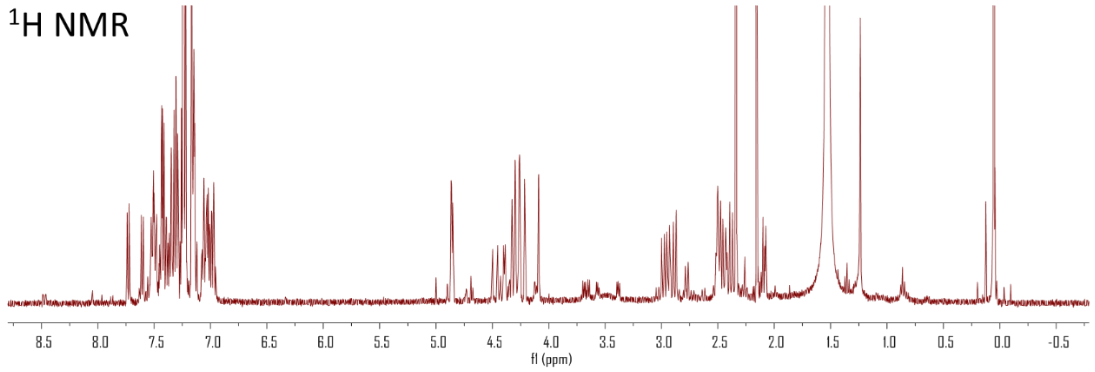

Figure S9: ¹H NMR spectrum of Fraction 7.

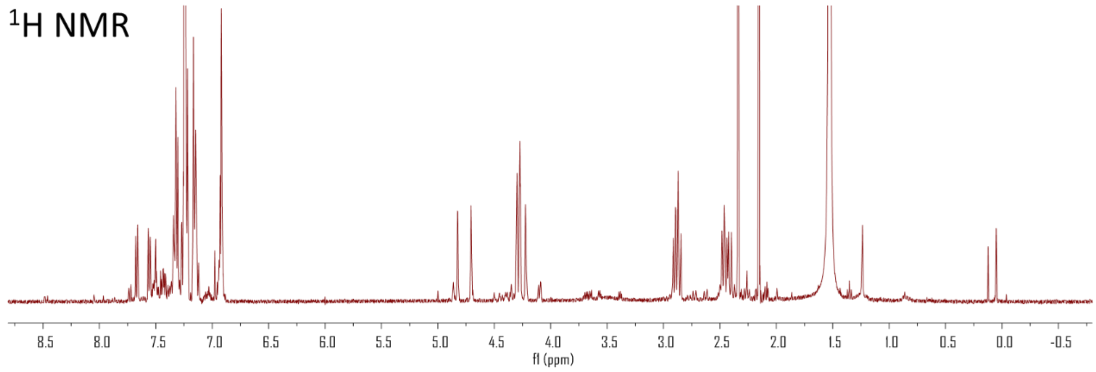

Figure S10: ¹H NMR spectrum of Fraction 8

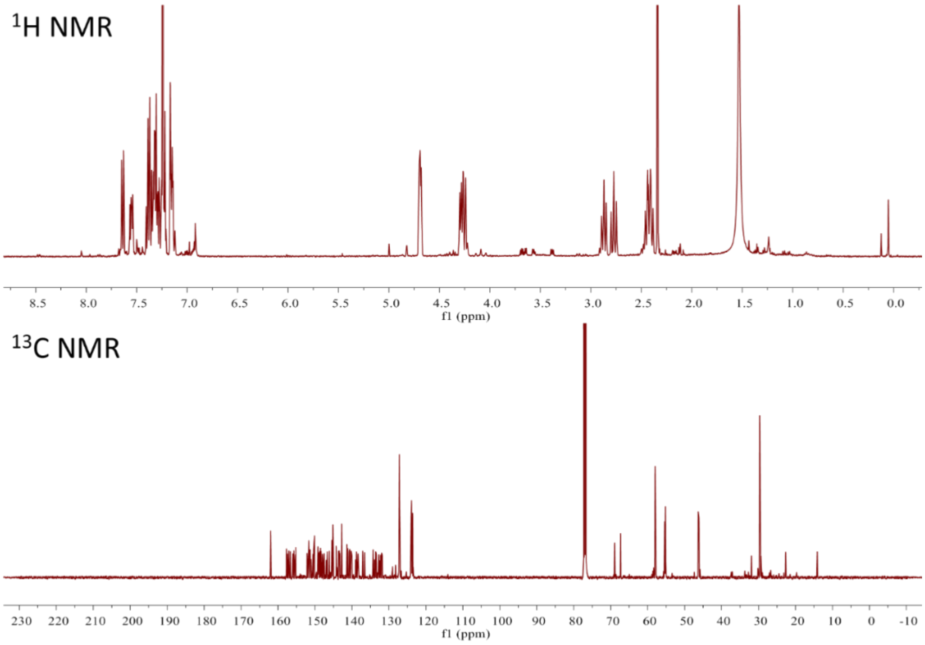

Figure S11: ^{1}H and ^{13}C NMR spectrum of Fraction 9-1.

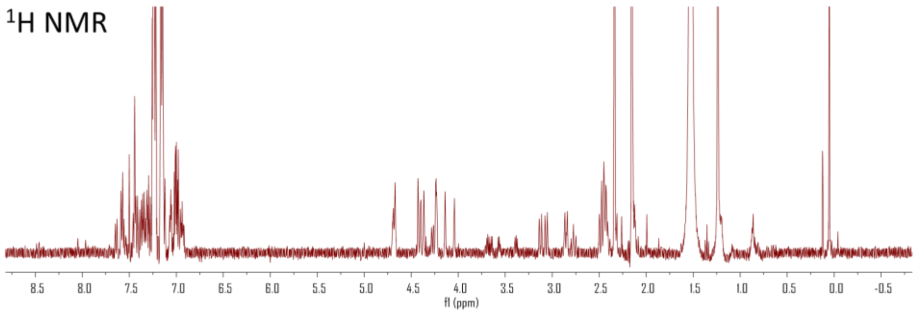

Figure S12: ^{1}H NMR spectrum of Fraction 10.

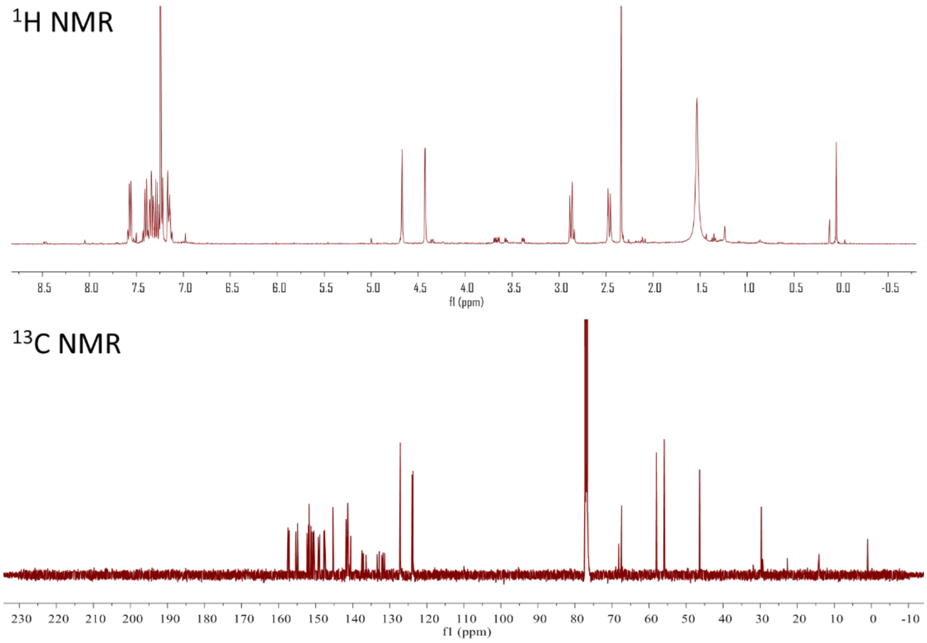

Figure S13: ^{1}H and ^{13}C NMR spectrum of Fraction 11.

UV–vis spectrum of $IC_{70}BA$ fractions 5 to 8

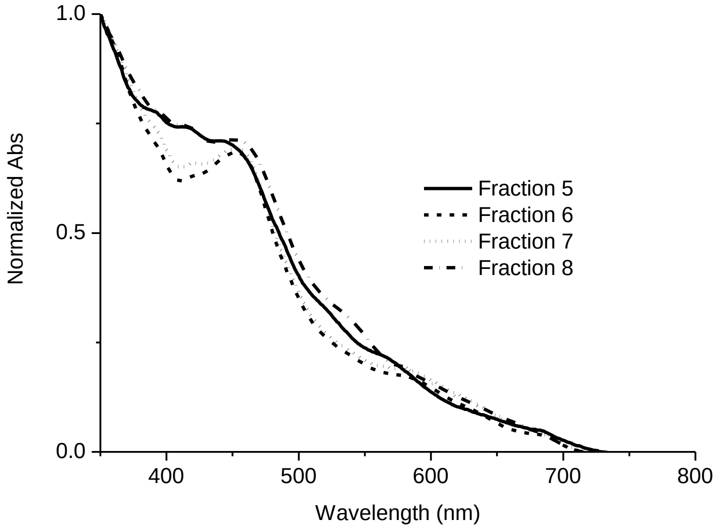

Figure S14: UV–vis spectrum of IC70BA fractions 5 to 8.

Electrochemical properties

The CVs were measured in CH_2Cl_2/MeCN 9:1 with 0.1 M TBA PF_6 at a scan rate of 50 mV/s and ferrrocene was added in the system as the internal reference. The reference electrode was a silver wire Ag/Ag^+ pseudo reference, the counter electrode was platinum and the working electrode was glassy carbon.

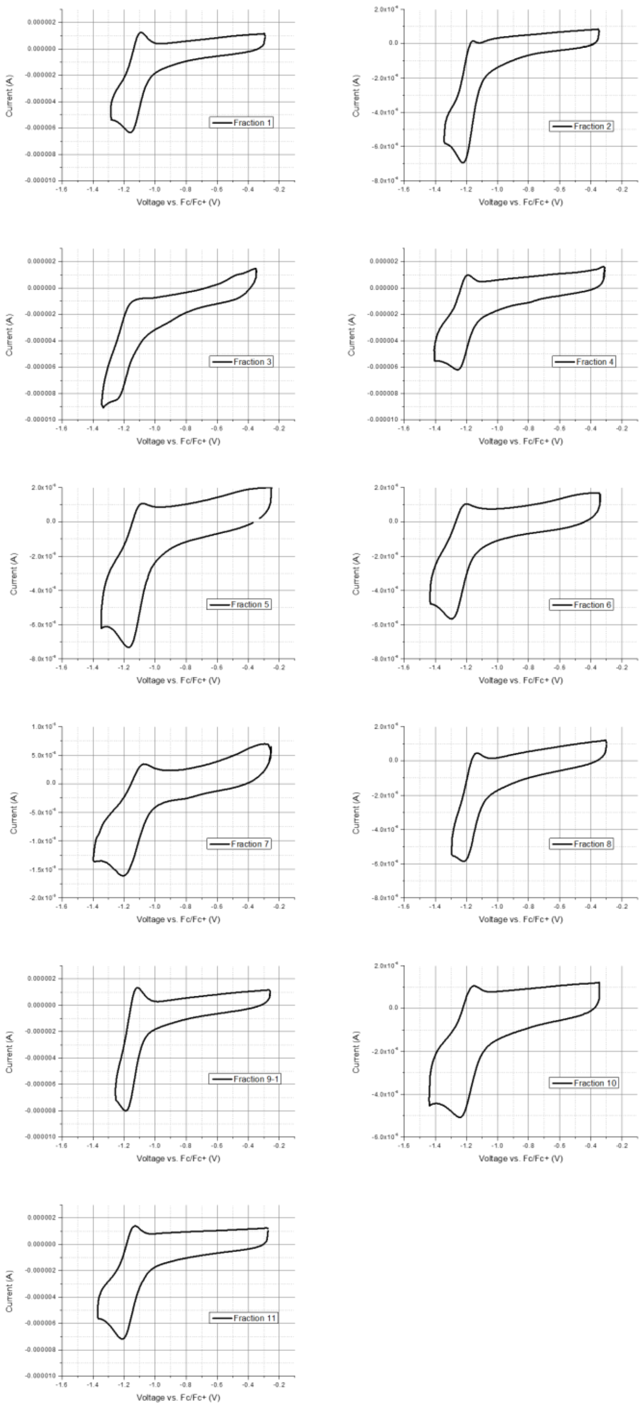

Figure S15: Cyclic voltammogram of all $IC_{70}BA$ fractions.

Device fabrication and characterization

Bulk heterojunction solar cell devices were fabricated and characterized as described in our previous publication.[1] Details are provided here for convenience.

Poly(3,4-ethylenedioxythiophene):poly(styrenesulfonate) (PEDOT:PSS; Baytron AI 4083 from HC Starck, 30 nm) were deposited by spin coating on patterned glass substrates which were washed by acetone, and 2-propanol in an ultrasonication bath and UV/ozone-treated. The PEDOT:PSS films were baked at 140 °C for 10 min in air. An active layer of the device consisting of the blend of P3HT and $IC_{70}BA$ with a ratio of 1:1 was then spin coated from *o*-dichlorobenzene solvent (20 mg/mL) with a thickness of ≈ 145 nm. The device was solvent annealed for 10 min followed by thermal annealed at 150 °C for 10 min in glove box. The films were transferred to a metal evaporation chamber and a bilayer cathode consisted of Ca (20 nm) capped with Al (100 nm) was deposited through a shadow mask (active area was 0.1 cm^2) at approximately 1×10^{-6} torr. Film thickness was determined by Veeco Dektak 150+Surface Profiler. The current density–voltage measurements of the devices were carried out using a 1 kW Oriel solar simulator with an AM 1.5G filter as the light source in conjunction with a Keithley 2400 source measurement unit. Solar measurements were carried out under 1000 W/m^2 AM 1.5G illumination conditions. For accurate measurement, the light intensity was calibrated using a reference silicon solar cell (PVmeasurements Inc.) certified by the National Renewable Energy Laboratory. Device fabrication and characterizations were performed in an inert environment without any encapsulation. Tapping-mode AFM (NanoScope II, Dimension, Digital Instrument Inc.) was carried out with commercially available tapping mode tips.

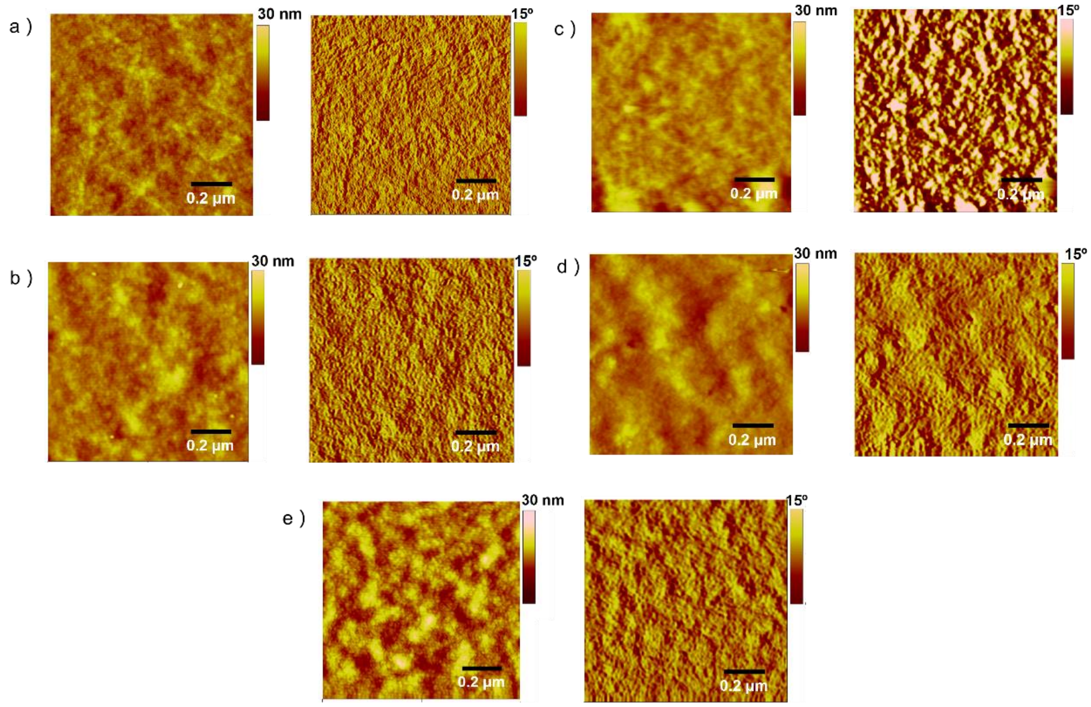

Figure S16: Tapping mode atomic force microscopy (AFM) height and phase images of the blend of P3HT and $IC_{70}BA$ a) Fraction 2, b) Fraction 3, c) Fraction 4, d) Fraction 9-1 and e) Fraction 11.

References

1. Wong, W. W. H.; Subbiah, J.; White, J. M.; Seyler, H.; Zhang, B.; Jones, D. J.; Holmes, A. B., Single Isomer of Indene-C70 Bisadduct—Isolation and Performance in Bulk Heterojunction Solar Cells. *Chem. Mater.* **2014,** 26, 1686-1689.
